# Knowledge, Awareness, and Perceived Attitude of Biomedical Waste Management Among Healthcare Personnel

**DOI:** 10.7759/cureus.73492

**Published:** 2024-11-11

**Authors:** Mrunal D Pawar, Kamala K A

**Affiliations:** 1 Pediatric and Preventive Dentistry, School of Dental Sciences, Krishna Vishwa Vidyapeeth (KVV), Karad, IND; 2 Oral Medicine and Radiology, School of Dental Sciences, Krishna Vishwa Vidyapeeth (KVV), Karad, IND

**Keywords:** attitude, awareness, biomedical waste, biomedical waste management, knowledge, practice

## Abstract

Introduction

Biomedical waste (BMW) management is a critical issue due to the hazardous nature of wastes generated daily in healthcare settings. Proper handling, which includes characterization, quantification, segregation, transport, and treatment, is vital to prevent risks to healthcare personnel, sanitation workers, and the general public. This study aimed to evaluate and compare the knowledge, awareness, and perceived attitude of BMW management among healthcare personnel in Karad City and its surrounding regions, focusing on dentists, general physicians, and nursing staff.

Materials and methods

A cross-sectional study was conducted using a structured questionnaire distributed to 150 healthcare staff members, including 50 dentists, 50 general physicians, and 50 nurses. Written consent was obtained from all participants prior to the study. The questionnaire consisted of 18 questions designed to assess the knowledge, awareness, and perceived attitude toward BMW management. The responses were analyzed statistically to determine the level of knowledge, awareness, and perceived attitude among the participants.

Results

The study found that the physicians had a significantly higher mean score for knowledge, awareness, and perceived attitude toward BMW management compared to the nurses and dentists, with a statistically significant difference (p < 0.05). Among the three groups, the nurses had higher scores for knowledge and awareness, while the dentists had a more favorable perceived attitude. The study also observed a disparity in knowledge regarding the appropriate storage time of BMW, with less than 50% of healthcare personnel aware of the correct duration. This finding contrasts with previous studies that reported higher levels of awareness.

Conclusion

The study underscores the necessity for continued education and training in BMW management for healthcare professionals. Improving awareness and adherence to proper waste management practices is essential to mitigate risks to human health and the environment. The differences in knowledge, awareness, and perceived attitude among dentists, physicians, and nurses suggest the need for targeted interventions to address specific gaps in knowledge and practices.

## Introduction

Biomedical waste (BMW) is a very unique type of trash that is produced daily in India and includes dangerous and toxic materials [1]. Its management employs a cradle-to-grave methodology, which involves characterizing, quantifying, transporting, separating, and treating BMWs [2]. Improper disposal poses significant risks to healthcare workers, sanitation personnel, and the general population.

Healthcare facilities generate substantial amounts of wastes, which include sharp wastes, cytotoxic materials, and anatomical wastes. Notably, only 10%-25% of BMWs is classified as hazardous, which can adversely impact the environment and ecological stability [3]. Dental practices, in particular, contribute significantly to the generation of wastes, much of which may be tainted with bodily fluids. This includes items such as cottons, plastics, latex, and glasses, along with small quantities of hazardous materials like mercuries, silver amalgams, and various chemical solvents [4]. Improper segregation, storage, and disinfection of these wastes can lead to the spread of infectious diseases, including HIV and hepatitis B and C [5].

To mitigate these risks, it is essential to establish biological waste treatment facilities in hospitals, nursing homes, clinics, dispensaries, veterinary practices, and pathology labs. It is a shared social responsibility to manage BMWs safely to protect both the environment and public health. In India, approximately 0.33 million tons of wastes is generated, with 10%-25% considered hazardous. Challenges in effective hospital waste management stem from a lack of awareness, motivation, knowledge, and financial resources [6]. Inadequate concern, drive, knowledge, and financial considerations are a few of the issues with appropriate hospital waste management [7]. Teaching institutions are essential to the healthcare system, as they train future healthcare professionals and all individuals involved in community caregiving. Studies from various regions of the country continue to show gaps in knowledge, deficiencies in attitudes, and inconsistencies in practices among healthcare professionals, which are concerning issues. This study aims to assess and enhance the awareness, knowledge, and perceived attitude of medical staff in Karad City and its surrounding places, ultimately promoting greater understanding among those less informed.

## Materials and methods

A cross-sectional study was conducted over a one-year period, from January 2019 to 2020, at Krishna Vishwa Vidyapeeth (Deemed to be University), Karad.

Data were gathered through a self-administered questionnaire, which was pretested to ensure clarity and to confirm that it effectively addressed all relevant topics for this research study. This step focused on evaluating the validity of the contents of the questionnaire. Participants in the pretesting phase included department heads of the study hospital, such as dentists, physicians, and nurse practitioners. The pretesting took place at the same hospital, but those involved in this phase were excluded from participating in the actual study. Results from the pretesting indicated that all respondents were satisfied with the questionnaire, affirming its adequacy and relevance to the study's objectives.

This questionnaire was then distributed to 150 staff members. The study population included 50 dentists, 50 general physicians, and 50 nursing staff. Written consent was obtained from all the participants before they were given the questionnaire. Eighteen questions were included in the proforma (Appendices), and the percentage of the correct and incorrect answers for each question was obtained and statistically analyzed.

## Results

Data were acquired using a self-administered questionnaire. The questionnaire was pretested to ascertain ease of understanding and to determine if it was worded to elicit all the materials of interest for this research study. Therefore, this process was concerned with assessing the validity of the contents of the questionnaire.

Participants in the pretesting phase included department heads of the study hospital, such as dentists, physicians, and nurse practitioners. This pretesting was carried out at the same hospital; however, those involved in the pretesting were excluded from the actual study. The results from this phase indicated that all respondents were satisfied, confirming that the questionnaire was appropriate and relevant to the study's aims.

The data were coded and double-entered into a relational database using Microsoft Excel (Microsoft Corp., Redmond, WA). The data entry subject was designed to check referential integrities, identify missing values, and ensure adherence to acceptability restrictions. Errors identified at any level were referred back to the field for correction. The percentages and their 95% confidence intervals (CIs) are presented. Data analysis was conducted using IBM SPSS Statistics for Windows, Version 22.0 (Released 2013; IBM Corp., Armonk, NY). Results were summarized using percentages, means, standard deviations, and 95% CIs. The Kruskal-Wallis test was employed to compare groups, with statistical significance set at p < 0.05.

Table 1 presents the profiles of the respondents. Of the 150 participants, 91 (60.67%) were male and 59 (39.33%) were female respondents. Regarding educational qualifications, 78 (52%) had completed undergraduate degrees, while 72 (48%) had postgraduate degrees. In terms of experience, 47 (31.33%) of the respondents had up to five years of experience, whereas 103 (68.67%) had more than five years. Among the 150 participants, 33.33% were nurses, general physicians, and dentists, among others.

**Table 1:** Profile of the respondents

Demographic variables	Frequency (%)
Gender	
Male	91 (60.67%)
Female	59 (39.33%)
Educational qualification	
Undergraduates	78 (52%)
Postgraduates	72 (48%)
Healthcare experience	
Up to five years	47 (31.33%)
More than five years	103 (68.67%)
Occupation	
Nurse	50 (33.33%)
General physician	50 (33.33%)
Dentist	50 (33.33%)

**Figure 1:**
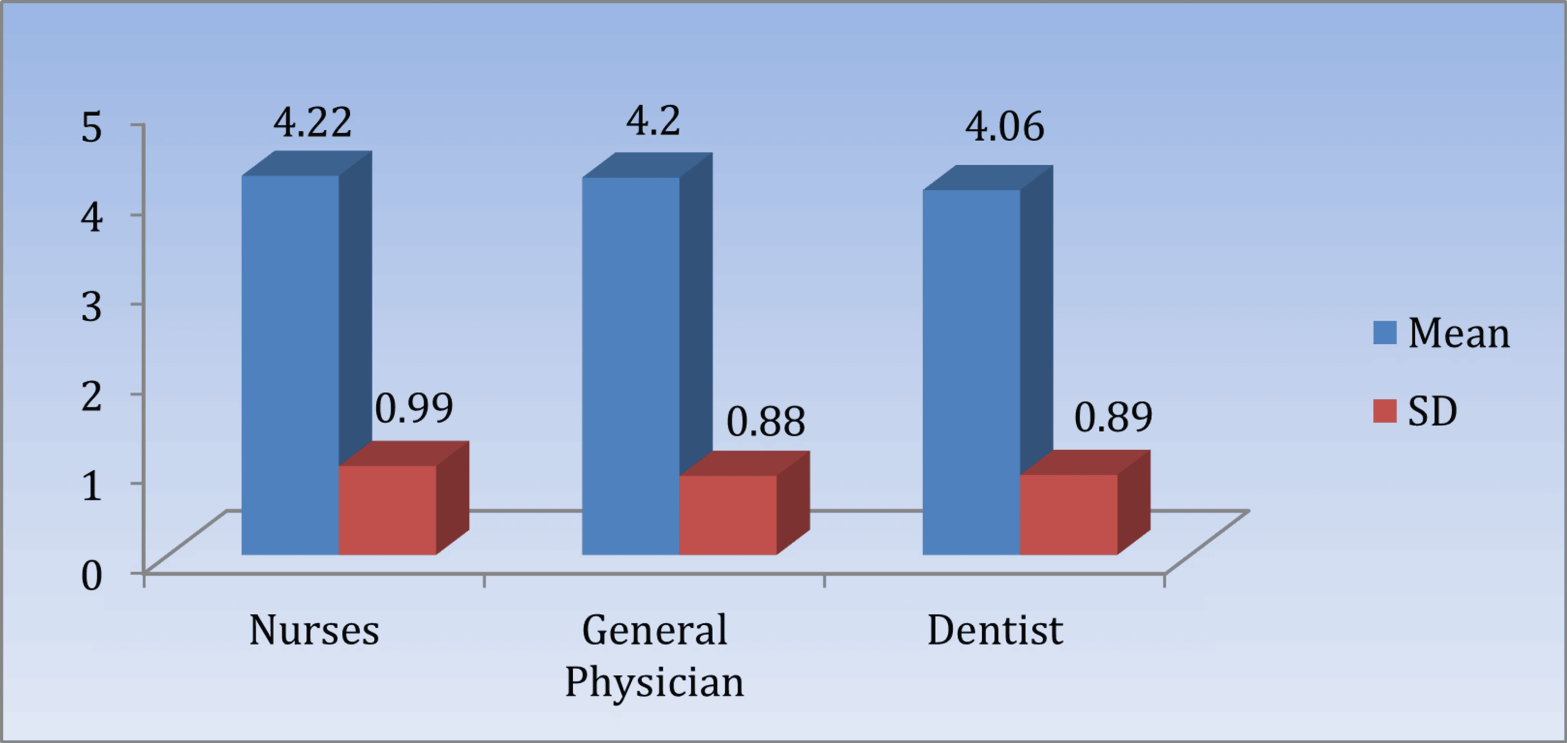
Means and SDs of the knowledge scores regarding the practice of biomedical waste management among respondents

Table 2 and Figure 1 present the mean knowledge scores of BMW management among nurses, general physicians, and dentists, significant at p > 0.05.

**Table 2:** Comparison of the mean knowledge scores regarding the practice of biomedical waste management among respondents (n = 150) Nurses vs. dentists: t = 0.88, p = 0.38. GPs vs. dentists: t = 0.82, p = 0.41. p < 0.05 is considered statistically significant. GPs: general physicians.

Knowledge	Mean	SD	95% CI		KW value	p-value
Nurses	4.22	0.99	3.93	4.5	1.373	0.5034
General physicians	4.2	0.88	3.94	4.45		
Dentists	4.06	0.89	3.81	4.3		

**Figure 2:**
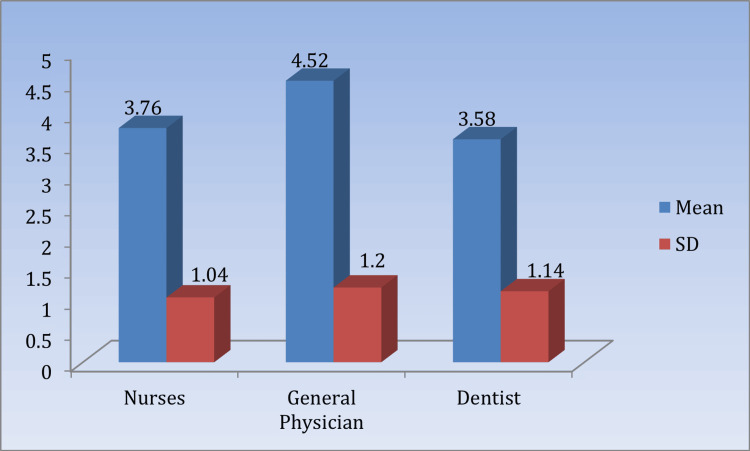
Means and SDs of the awareness scores regarding the practice of biomedical waste management among respondents

Table 3 and Figure 2 present the mean awareness scores of BMW management among nurses, general physicians, and dentists, significant at p < 0.05.

**Table 3:** Comparison of the mean awareness scores regarding the practice of biomedical waste management among respondents (n = 150) Nurses vs. dentists: t = 0.82, p = 0.41. GPs vs. dentists: t = 4.02, p = 0.0001*. *Significant at p < 0.05. GPs: general physicians.

Awareness	Mean	SD	95% CI		KW value	p-value
Nurses	3.76	1.04	3.46	4.06	17.868	0.0001*
General physicians	4.52	1.2	4.18	4.86		
Dentists	3.58	1.14	3.25	3.9		

**Figure 3:**
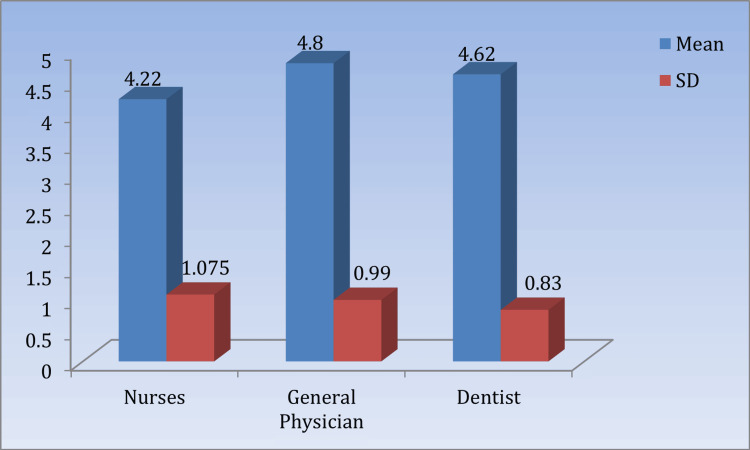
Means and SDs of the perceived attitude scores regarding the practice of biomedical waste management among respondents

Table 4 and Figure 3 present the mean perceived attitude scores toward BMW management among nurses, general physicians, and dentists, significant at p < 0.05.

**Table 4:** Comparison of the mean perceived attitude scores regarding the practice of biomedical waste management among respondents (n =150) Nurses vs. dentists: t = 2.08, p = 0.0399*. GPs vs. dentists: t = 0.99, p = 0.33. *Significant at p < 0.05. GPs: general physicians.

Perceived attitude	Mean	SD	95% CI		KW value	p-value
Nurses	4.22	1.075	3.91	4.53	8.446	0.0147
General physicians	4.8	0.99	4.52	5.08		
Dentists	4.62	0.83	4.38	4.86		

## Discussion

In India, Maharashtra is among the top five BMW-generating states. As of July 2020 in India, the highest volume of BMW is produced in south-western Maharashtra, which was significantly increased by the pandemic, with 17.5 metric tons per day.

Given the harmful impacts on the human health and the environment, numerous studies from developing countries have highlighted the lack of knowledge and awareness among healthcare personnel. This observational study aimed to examine and compare the levels of knowledge, awareness, and perceived attitude of dentists, general physicians, and nurses.

Among the 150 respondents, 91 (60.67%) were male participants, while 59 (39.33%) were female participants. In terms of educational qualifications, 78 (52%) had completed undergraduate degrees, and 72 (48%) held postgraduate degrees. Additionally, 47 (31.33%) of the respondents had up to five years of experience, whereas 103 (68.67%) had more than five years of experience.

Knowledge regarding BMW management is essential due to its infectious nature. When asked about the storage time of wastes, less than 50% of the healthcare personnel were unaware about it. This was totally opposite to the study conducted by Patnaik and Sharma in Bhubaneswar, where 93.2% of dentists and 82% of nurses where aware of how long BMWs can be stored [7]. Section 1 of the proforma consisted of questions testing the knowledge of BMW among dentists, nurses, and general physicians, which was almost the same, with p > 0.05. Thirty-four percent of the total respondents had excellent knowledge of BMW management, which was comparatively higher than the results reported by Sharma et al., which was 30% [8]. In this study, the general physicians had comparatively high awareness of BMW management, followed by the nurses and dentists. In response to the questions asked regarding color-coding for the disposal of sharp wastes, 70% of the dentists and general physicians were aware of the use of white or blue container in disposing sharp wastes, followed by 60% of the nurses, which was strange. The awareness about color-coding for sharp wastes among the dentists in this study was 70%, which was higher than a similar study conducted by Kumar and Rahman [9]. 

Seventy-six percent of the dentists agreed to the fact that waste management is a teamwork, which was comparatively less than the findings of Bala and Narwal, accounting for 92% [10]. According to Sharma et al., 65% of the respondents agreed that waste management required a teamwork [8].

## Conclusions

While comparing the levels of knowledge, awareness, and perceived attitude, it was observed that the mean knowledge score regarding BMW management was good among the general physicians, compared to the nurses and dentists, and it was found to be statistically significant at p < 0.05 for awareness and perceived attitude. Also, it was observed that the mean knowledge and awareness scores regarding BMW management among nurses were higher than the scores among the dentists, whereas, vice versa, the perceived attitude score regarding BMW management among dentists was higher than the score among the nurses.

## Appendices

Proforma

Tick the appropriate answer.

Occupation

* General physician 

* Dentist

* Nurse

Section 1: Knowledge of BMW Management, Hazards, and Legislation

1. Do you know about BMW generation and legislation?

a) Yes

b) No

c) Not sure

2. What agencies regulate wastes generated in healthcare centers?

a) State

b) Private

c) Don't know

3. Do you think it is important to know about BMW generation, hazards, and legislation?

a) Yes

b) No

c) Somewhat

4. Which statement describes that one type of BMW?

a) Materials that may be poisonous, toxic, or flammable and do not pose disease-related risk

b) Wastes that are saturated to the point of dripping with blood or body fluids contaminated with blood

c) Wastes that don’t pose a disease-related risk

5. According to BMW management rules, wastes shouldn’t be stored beyond?

a) 12 hours

b) 48 hours

c) 72 hours

d) 96 hours

6. Do you need a separate permit to transport BMWs?

a) Yes

b) No

c) Cannot say

Section 2: Level of Awareness Regarding BMW Management Practices

1. Do you know about the color-coding segregation of BMWs?

a) Yes

b) No

c) Not sure

2. Do you follow the color-coding for the disposal of BMWs?

a) Yes

b) No

c) Not sure

3. Documents with confidential patient information are to be disposed of into the paper-recycling bins.

a) True

b) False

c) Don’t know

4. The color code for BMWs to be autoclaved and disinfected is?

a) Red

b) Black

c) Yellow

d) Blue/white

5. Sharp wastes should be disposed in a?

a) Yellow container

b) Red container

c) White/blue container

d) Don’t know

6. Gloves, gauze pieces, and cottons should be disposed in a?

a) Yellow container

b) Red container

c) White/blue container

d) Don’t know

Section 3: Attitude/Behavior Assessment Toward BMW

1. Safe management of healthcare wastes is never an issue at all.

a) Agree

b) Disagree

c) Cannot comment

2. Waste management is a teamwork; no single class of people is responsible for its safe management.

a) Agree

b) Disagree

c) Cannot comment

3. Efforts on the safe management of wastes increase the financial costs of hospitals.

a) Agree

b) Disagree

c) Cannot comment

4. Safe management of wastes generated in healthcare centers is an extra burden to the workload.

a) Agree

b) Disagree

c) Cannot comment

5. Have you attended programs that enhance your knowledge about waste management?

a) Yes

b) No

c) Cannot comment 

6. Do you think that the infectious wastes should be sterilized by autoclaving before shredding and disposing?

a) Yes

b) No

c) Cannot comment

## References

[1] Uzych L (1990). Medical waste management. Regulatory issues and current legal requirements. J Environ Health.

[2] Das SK, Biswas R (2016). Awareness and practice of biomedical waste management among healthcare providers in a tertiary care hospital of West Bengal, India. Int J Med Public Health.

[3] Goyal S, Dileep CL, Mathur A, Chaudary S, Makkar K, Battra M, Sood P (2015). Knowledge, attitude and practices regarding biomedical wastes among health care professionals in Sri Ganganagar city: a cross-sectional study. DHR-IJMS.

[4] Rasheed S, Iqbal S, Baig LA, Mufti K (2005). Hospital waste management in the teaching hospitals of Karachi. J Pak Med Assoc.

[5] Datta P, Mohi GK, Chander J (2018). Biomedical waste management in India: critical appraisal. J Lab Physicians.

[6] Dhanya RS, Betur AP, Bulusu A, Vj A, Koshy PV, Pinto B (2016). Management of biomedical waste in dental clinics. Int J Oral Care Res.

[7] Patnaik S, Sharma N (2018). Assessment of cognizance and execution of biomedical waste management among health care personnel of a dental institution in Bhubaneswar. J Indian Assoc of Public Health Dent.

[8] Sharma A, Sharma V, Sharma S, Singh P (2013). Awareness of biomedical waste management among health care personnel in Jaipur, India. Oral Health Dent Manag.

[9] Kumar SP, Rahman R (2017). Knowledge, awareness, and practices regarding biomedical waste management among undergraduate dental students. Asian J Pharm Clin Res.

[10] Bala S, Narwal A (2013). Awareness of bio-medical waste management among dental college and hospital employees-a panoramic view. Int J Oral Health Comm Dent.

